# More than blood: app-tracking reveals variability in heavy menstrual bleeding construct

**DOI:** 10.1186/s12905-023-02312-4

**Published:** 2023-04-11

**Authors:** Amanda A. Shea, Fiorella Wever, Cécile Ventola, Jonathan Thornburg, Virginia J. Vitzthum

**Affiliations:** 1 Clue by BioWink GmbH, Adalberstrasse 7-8, 10999 Berlin, Germany; 2 University of Amsterdam, Amsterdam, Netherlands; 3 Indiana University, Bloomington, IN USA

**Keywords:** Heavy menstrual bleeding, Period heaviness, Menstrual health, Period tracking, Mobile application, Mhealth, Bleeding volume, Quality of life, Period-associated impairments, Recall bias

## Abstract

**Background:**

Heavy menstrual bleeding (HMB) is associated with impaired quality of life and may signal serious health problems. Unresolved challenges in measuring menstrual bleeding and identifying HMB have hampered research and clinical care. Self-reported bleeding histories are commonly used but these may be influenced by recall bias, personal beliefs regarding “normal” flow volume, and the experience of other physical symptoms or disruptions to daily life. The potential usefulness of menstrual-tracking mobile applications, which allow real-time user-entered data recording, for assessing HMB has not been studied. We evaluated recall bias in reported period duration, the relationship of tracked period duration and daily flow volume to subsequently reported period heaviness, variation in quality of life associated with increasing period heaviness, and the advantages and limitations of using app-tracked data for clinical and research purposes.

**Methods:**

An online questionnaire was distributed to current users of Clue, a commercially available menstrual health tracking app, asking them to characterize their last period. We compared responses to the user’s corresponding Clue app-tracked data. The study sample comprised 6546 U.S.-based users (aged 18–45 years).

**Results:**

Increasing reported heaviness was associated with increasing app-tracked period length and days of heavy flow, impaired quality-of-life (especially body pain severity), and disrupted activities. Of those reporting having had a heavy/very heavy period, ~ 18% had not tracked any heavy flow, but their period length and quality-of-life indicators were similar to those who had tracked heavy flow. Sexual/romantic activities were the most affected across all flow volumes. Compared to app-tracked data, 44% recalled their exact period length; 83% recalled within ± 1 day. Overestimation was more common than underestimation. However, those with longer app-tracked periods were more likely to underestimate period length by ≥ 2 days, a pattern which could contribute to under-diagnosis of HMB.

**Conclusion:**

Period heaviness is a complex construct that encapsulates flow volume and, for many, several other bleeding-associated experiences (period length, bodily impairments, disruptions of daily activities). Even very precise flow volume assessments cannot capture the multi-faceted nature of HMB as experienced by the individual. Real-time app-tracking facilitates quick daily recording of several aspects of bleeding-associated experiences. This more reliable and detailed characterization of bleeding patterns and experiences can potentially increase understanding of menstrual bleeding variability and, if needed, help to guide treatment.

**Supplementary Information:**

The online version contains supplementary material available at 10.1186/s12905-023-02312-4.

## Background

Our objective in this study is to contribute to the nascent body of scientific inquiry into menstrual bleeding from the individual’s perspective, with the larger goal of improving menstrual health. More specifically, we investigate the suite of factors (e.g., bleeding volume, bleeding duration, and the impacts of bleeding on daily life) that individuals may be using to characterize any of their own periods as “heavy”.

Empirically based comprehensive knowledge of variability in menstruators’ bleeding experiences and how this variability is related to diagnoses and treatment of menstrual disorders is sorely needed. In the United States alone, it’s estimated that 6.5 to 20 million women of reproductive age are negatively impacted by heavy menstrual bleeding (HMB) [1]. In addition to the significant personal impacts, costs of treatment and lost productivity run to several billion dollars per year [1, 2].

Estimates of the prevalence of HMB vary greatly (from 9 to 52%), depending on the assessment method and population [1, 3]. These disparities arise in part from several challenges in assessing an individual’s personal experience of menstrual bleeding. A commonly used clinical criterion is menstrual blood loss of > 80 mL per cycle [4, 5], but blood volume is cumbersome to measure. Collecting and storing used sanitary products for later laboratory analysis is impractical for most women [6], and currently available pictorial blood loss assessment charts have been found to be unsuitable for use other than in controlled clinical studies [7]. Rather than specifying quantities of flow volume, ACOG (American College of Obstetricians and Gynecologists), in its public facing website [8], lists several proxy indicators of copious bleeding (e.g., needing to change pads or tampons during the night), any one of which may indicate HMB.

Another commonly used approach in HMB screening is self-reported bleeding history [9]. However, intermittent symptoms, like those associated with HMB, are vulnerable to recall bias. Also, bleeding history questionnaires may request, or unintentionally elicit, only summary information that fails to represent variability in day-to-day experiences [10].

In addition, period “heaviness” has both common and clinical meanings, but the differences between these usages are under-explored. This gap in common understanding can contribute to client dissatisfaction, misdiagnoses, over- or undertreatment, and wasted resources [11]. Not all persons who report that they have heavy bleeding meet clinically defined bleeding criteria for HMB. For example, in a study using the (gold standard) alkaline hematin method to measure blood loss, only 26% of those describing their period as heavy exceeded the 80ml threshold [12]. This and similar findings [11, 13] suggest that at least some persons consider their periods to be heavy based on factors other than just flow volume (e.g., period length, pain severity, missed activities). Recognizing this, health care providers (HCPs) have begun to incorporate measures of the impact of menstrual bleeding on quality of life into their diagnosis and treatment of HMB. For example, the UK National Health Services’ initial screening includes “[do you] avoid daily activities, like exercise, or take time off work because of your periods” in its list of HMB indictors [14]. The International Federation of Gynaecology and Obstetrics (FIGO) explicitly acknowledges the centrality of impacts on quality of life, defining HMB as “an excessive menstrual blood loss that interferes with the woman’s physical, social, and material quality of life” [15]. Although various bleeding-specific quality-of-life instruments have been proposed and used, there is no single standard instrument [16–19].

A major challenge in the development and use of criteria for identifying HMB is the marked variability between persons in their menstrual biology *and* in their personal understanding and experience of menstrual bleeding. Each menstruating adult has a sense of their own “normal” menstrual cycle [20] and the attributes of their cycle (period and cycle duration, flow volume, physical pain, disrupted activities) that are most salient to them. Deviations from personal bleeding norms may be perceived negatively (i.e., as cause for concern about health or fertility) even if the change is small by clinical criteria [11, 21, 22]. Shared beliefs, knowledge and language (within families, among friends, and in the fabric of the wider culture) will also shape individual assessments of one’s own body and any deviations in its functioning [23].

Thus describing a period as “long” or “heavy” may reflect several aspects of the experience of bleeding including volume, duration, impaired physical or emotional wellness, and differences from one’s own usual pattern or normative cultural concepts. O’Flynn and Britten [11] persuasively argued for the need to “engage more fully with the [menstruating] patient’s definition and experience.” Based on meta-ethnography analysis that drew on their [11] work, Garside and colleagues [13] proposed a model of menstrual experience that addressed some of the same points. However, these insights, principally gained from interviews of modest samples of clinical patients, have not yet been evaluated in large non-clinical samples.

The increasing popularity of menstrual tracking mobile applications (“apps”), used by a third of U.S. women to catalog their daily bleeding experiences in real-time [24], facilitates collection of granular longitudinal data from a very large and diverse non-clinical sample. This technology affords a novel opportunity to gain empirically grounded insights into menstruating persons’ characterizations of their individual menstrual experiences.

In this study we utilized de-identified user-tracked data from Clue, a widely used menstrual health tracking app [25], compared individuals’ app-tracked bleeding data to their responses to an on-line questionnaire about their last completed period, and assessed the correspondence of these two data sources. To better understand the multi-faceted individual experience of menstrual bleeding, we also examined the relationships of tracked period duration, tracked daily flow volume, and reported quality of life indicators to a respondent’s self-assessment of their last period’s heaviness. To our knowledge, this is the first study to address these questions using real-time app-tracked data from a non-clinical sample of more than 6500 persons.

## Methods

### Study population

This is a comparative observational study. Data recorded in real time by the study participants were compared to reported data provided by the same respondents in a subsequent questionnaire.

An online questionnaire, in either English or Spanish depending on the app’s language setting, was distributed via in-app messages to current U.S. users, aged 18 years and older, of Clue < helloclue.com> [25], a menstrual health tracking app that has been used to collect data for other studies of menstrual cycling [26]. Data were collected from November 9, 2020 through December 1, 2020. Each respondent gave explicit informed consent for the integration of their questionnaire responses (hereafter referred to as “reported” data) with the individual menstrual cycle data that each had tracked in the Clue app (hereafter referred to as “app-tracked” data) during the months prior to receiving the questionnaire. Respondents were informed that the survey was for the purpose of scientific research and were asked to explicitly affirm that they had taken the questionnaire seriously; those who didn’t affirm were excluded from analyses.

To evaluate the generalizability of these findings to the U.S. population of adult women (as reported in the 2020 Census), study participants were asked their age and a multiple-selection optional question on their ethnic background.

### App-tracked data collection

**Fig. 1:**
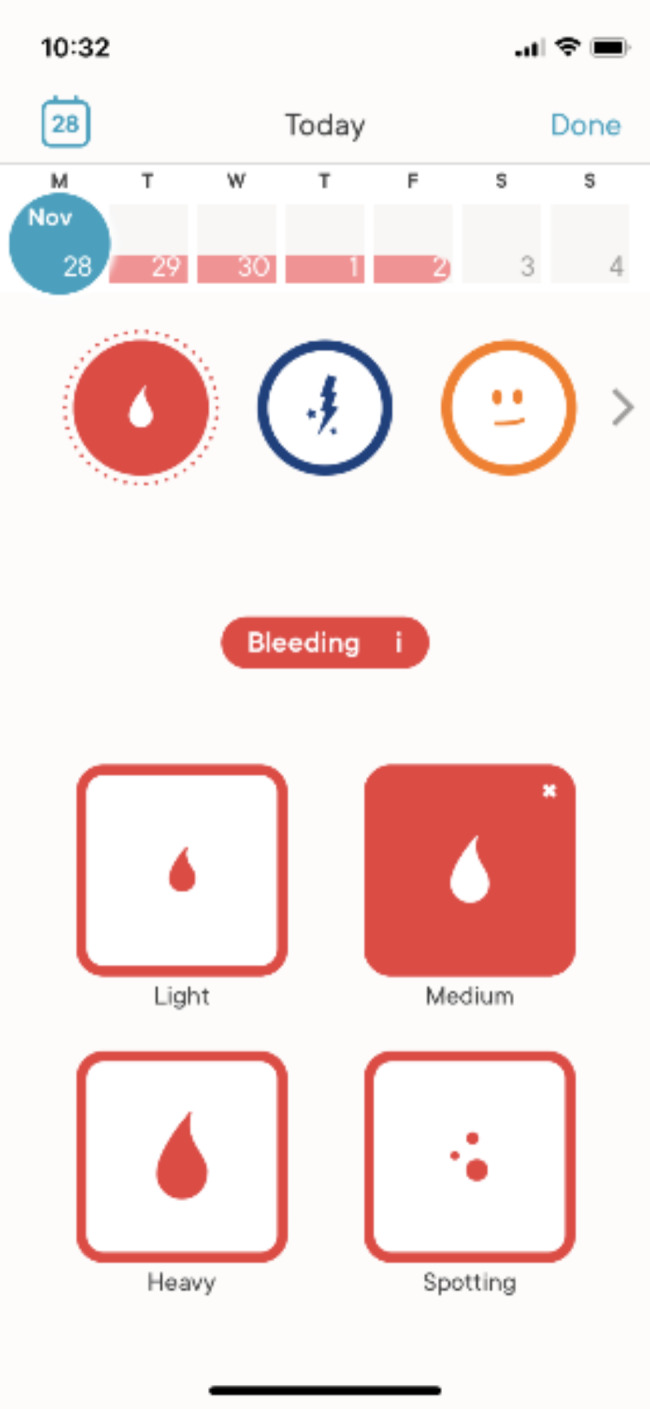
Screen in the Clue app for recording daily bleeding volume (©Clue by Biowink GmbH, Berlin, Germany)

Clue users can track their period by selecting one of four bleeding volumes (heavy, medium, light and spotting) for each day they experience bleeding (Fig. 1). Descriptions of each of the tracking options were provided within the app to support users in utilizing the options as intended. Users could access the information directly from the tracking screen by clicking on the ‘i’ (located mid-screen next to ‘Bleeding’), however, users do not always read the text or use it to inform their tracking behavior. For the present analyses, we used data specific to the most recent completed menstrual period tracked by the user that had occurred before the date on which the user submitted their questionnaire responses.

### Questionnaire data collection

Respondents were asked to characterize their last period (duration and heaviness), excluding spotting days, and its impact on their quality of life. Respondents were explicitly and repeatedly instructed that the questions pertained to the most recent previous period (regardless of whether they were/were not currently experiencing menstrual bleeding at the time of responding to the questionnaire).

The questions inquiring about the attributes of the last completed period were:

1. During your last completed period (if you are on your period right now, consider the previous one), how many days did you bleed for? Only include days where you would need to use a product such as a tampon, menstrual cup, or pad. Do not include spotting.
2. How heavy was your bleeding in your last completed period?
  Choose one: Very light, Light, Moderate, Heavy, Very heavy

Two questions that evaluated the impact of menstrual bleeding on the respondent’s quality of life asked about the severity of physical and emotional experiences related to menstruation (Question 3) and any need to forego common daily activities (Question 4).

(3) Score your experience for each of the following experiences on a scale of 5 options: None, Mild, Moderate, Severe, Very Severe [a Likert scale appeared under each question].
  3a How severe was your *body pain (e.g. cramps, back pain, breast pain*) in the week before or during your last completed period? Do not consider headaches or migraines.
  3b How severe were your *headaches or migraines* in the week before or during your last completed period?
  3c How severe was your *tiredness or difficulty sleeping* in the week before or during your last completed period?
  3d How severe were your *digestive symptoms (e.g. nausea, upset stomach, diarrhea, constipation)* in the week before or during your last completed period?
  3e How severe were your *emotional changes (e.g. sadness, depression, mood swings)* in the week before or during your last completed period?
(4) During your last completed period, which of the following were you not able to do at least once because of your period or period-related symptoms? Select all that apply.
  4a School or work
  4b Household tasks such as cooking, cleaning, or grocery shopping
  4c Traveling, hobbies, or other leisure activities
  4d Social activities with friends or family
  4e Religious activities or community events
  4f Sexual or other romantic activities
  4g My period did not prevent me from doing anything

### Exclusion criteria

Respondents were excluded from all analyses if (1) they did not answer questions 1 (on period length) and 2 (on period heaviness) above, (2) their prior period length tracked in the app was either one day or greater than ten days, or (3) their last cycle length tracked in the app was greater than 50 days. After these exclusions, the final analytical sample was 6546 respondents except for the analyses of reported period length, which was 6338 because 218 persons entered something other than a positive integer in response to question 1.

Users with an app-tracked last period length of one day (n = 16) were excluded from the analyses because some app users may occasionally or habitually track only the first day of bleeding regardless of the total period length. This tracking behavior may occur because the first bleeding day of each tracked cycle is sufficient information for the app to provide cycle length statistics and predict the start of the next cycle. Of the 16 excluded, one gave an unreadable answer for period length. The distribution of reported period lengths for the remaining 15 was 1 day (n = 1, 7% of 15), 2 days (n = 1, 7%), 3 days (n = 3, 20%), 4 days (n = 6,), 5 days (n = 2,), 6 days (n = 1, 7%), 7 days (n = 1, 7%). The distribution (median = 4 days) suggests that most of these users had likely tracked only their first day of bleeding.

All bleeding, whether tracked in the app as period bleeding or reported in response to survey questions regarding periods, is referred to here as period or menses, even though some contraceptive methods suppress or alter bleeding patterns [27]. For example, withdrawal bleeding that may occur during the hormone-free days of a combined oral contraceptive pill regime is often incorrectly perceived as a “period”. Nonetheless, because this study’s focus is the individual’s characterization of their own bleeding heaviness regardless of the possible cause of any heaviness, a person’s use of contraception was not an exclusionary criterion. Any Clue app users whose bleeding had ceased because of contraceptives or any other reason are unlikely to be in our study sample as they would not have been recently tracking bleeding and thus were less likely to have been using the app.

### Data analysis

Analyses were performed with Perl *v.* 5.32.1 and the Date::Pcalc library *v.* 6.1; Python *v.* 3.6.9 (Pandas *v.* 1.0.3 and Numpy *v.* 1.16.1); plots were made with Matplotlib *v.* 3.2.1 or Seaborn *v.* 0.11.1.

## Results

### Participant attributes

The study sample comprised 6546 U.S.-based Clue users, aged 18–45 years, median age = 26 years (see Additional_file_1.pdf for demographic statistics of study sample). About 72% had at least some post-secondary (i.e., post-high school) education; about 27% had a high school or equivalent diploma; <1% lacked a high school diploma. About 2% took the survey in Spanish, the rest in English. Education and language did not differ by age (< 26 years versus 26 + years). Furthermore, none of the findings reported below differed by age.

Responses to a question on ethnic background (multiple selection of listed options and a write-in option were all permissible responses) were aggregated into categories comparable to those published for the U.S. 2020 Census data (see Additional_file_1.pdf) [28]. Of all survey respondents, 67.4% self-identified as white, 7.9% as Black, 4.6% as Asian, 0.5% as Native American, Alaskan Native, Native Hawaiian or Pacific Islander, and 6.9% identified as two or more of these groups (referred to as “*race*” in the Census). Note that, as was done in the U.S. Census report, in our study those persons identifying as Hispanic/Latinx *ethnicity* and also identifying with one (or more) of the race groupings listed above were included in the counts for the race group. The proportion of those self-identifying as Hispanic or Latinx (17.8%) was similar to the U.S. Census (18.5%). However, in the present study sample, about 70% of Hispanics/Latinx did not identify with one of the race groups listed above, which accounts to some extent for the lower percentages in those groups compared to the U.S. Census. In addition, the study sample had a higher proportion of those who self-identified with more than one race group (6.9%) compared to the U.S. Census population (2.8%) [28].

Compared to their age peers, 29% reported their own health to be the same (i.e., average), 66% reported their own health to be good or very good, and 5% reported their own health to be bad or very bad. Neither age nor the mean number of app-tracked heavy, medium and light days of bleeding varied with reported health status (analyses not shown). A slightly larger proportion of those with reported heavy/very-heavy bleeding self-reported as having bad/very-bad health than did those with reported light/very-light bleeding (6.6% vs. 5.2%).

Regarding their current contraceptive method, 25% were using a hormonal method (injection, implant, hormonal IUD, birth control pill, contraceptive patch, vaginal contraceptive ring), 50% were using a non-hormonal method (condom, diaphragm, fertility awareness method, withdrawal, male sterilization, copper IUD, emergency contraception, lactational amenorrhea), 17% had not used any method in the previous three months, and 8% did not specify their current method. Hormonal method users were slightly younger than those not using a hormonal method (median = 24 years vs. 26 years).

### Cycle attributes

The length in days of the most recently completed cycle (defined as the first day of menstrual bleeding up to and including the day before the next first day of menstrual bleeding) was calculated from the app-tracked data for each respondent. Sample statistics (n = 6546, mean = 30.0, SD = 5.55, median = 29) were similar to those reported for other studies of cycle length in U.S. women [29]. Median cycle length was 28 days in hormonal contraceptive users and 29 days in those not using hormonal contraception; mode = 28 days in both subsamples.

### Period length recall bias

**Fig. 2:**
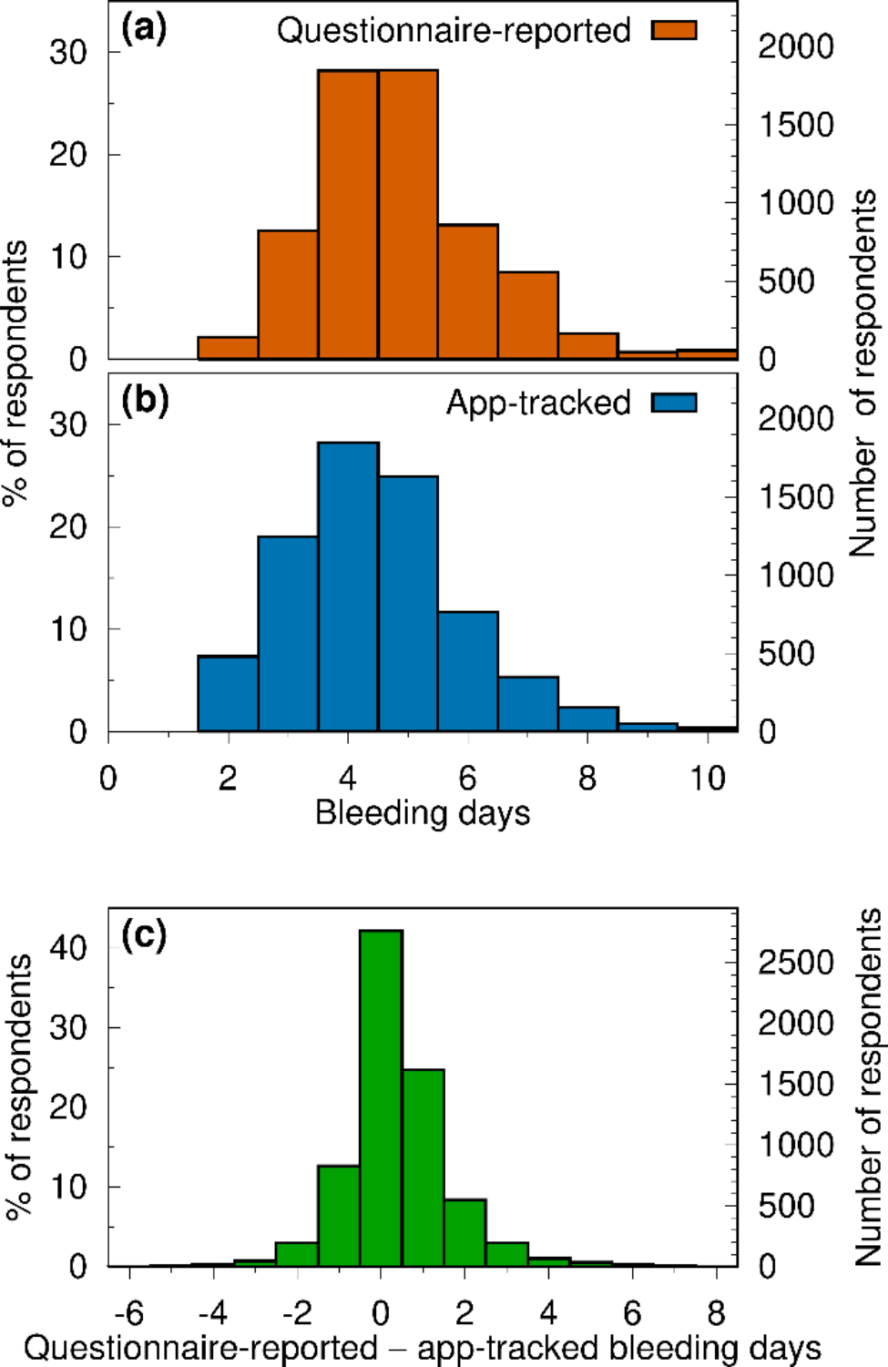
App-tracked vs. reported period lengths. (**a**) Histograms of reported (upper) and app-tracked (lower) total bleeding days in the most recently completed period [n = 6338]. (**b**) Histogram of the difference between reported and tracked period length (positive values = overestimation and negative values = underestimation of reported compared to tracked period length)

On average, reported period length was longer (median = 5, mode = 5, mean = 4.84, mean difference = 0.38, sd = 1.43, n = 6338, paired t = 24.3, p ≤ 0.0001) than users’ last completed app-tracked period length (median = 4, mode = 4, mean = 4.46, sd = 1.46, n = 6338). Specifically, 44% reported the same duration; 26% overestimated and 13% underestimated the tracked duration by one day (Fig. 2). In total, 83% reported the length of their previous period to within 1 day of the number of days tracked in the app. An additional 12% reported their previous period $\pm$2 days of the tracked duration. There was very little difference in tracked period length distribution between hormonal contraceptive users and those not using hormonal contraception. Fewer hormonal contraceptive users recalled their period length within one day than did those not using hormonal contraception (78% vs. 84%).

Period length recall bias varied with app-tracked period length. Table 1 presents the difference between reported period length and app-tracked period length, stratified by app-tracked period length. Those with a tracked period length of 4 to 7 days were most likely to report the same period length as they had tracked in the app (median and mode for the difference between reported and app-tracked period lengths are both 0). Those with shorter app-tracked periods were more likely to report a longer period length than had been tracked in the app (e.g., median difference of 2 days for those with 2-day periods), and those with longer app-tracked periods were more likely to report a shorter period than had been tracked in the app (median and mode differences of -2 days for those with 10-day app-tracked periods).

**Table 1:** Recall bias in period length (days), stratified by app-tracked period length (days)

App-tracked period length	N	Recall bias (Mean)	Recall bias (SD)	Recall bias (Median)	Recall bias (Mode)
2	461	1.78	1.47	2	1
3	1195	0.91	1.11	1	1
4	1798	0.47	1.01	0	0
5	1590	0.09	0.96	0	0
6	740	-0.12	1.15	0	0
7	339	-0.46	1.30	0	0
8	150	-0.71	1.39	-1	0
9	45	-1.31	1.61	-1	-2
10	20	-1.70	1.49	-2	-2

Recall bias = reported period length minus app-tracked period length (total N = 6338).

Increasing reported period heaviness was associated with increasing mean app-tracked period length (Table 2). Median and mode app-tracked period length were longer at higher levels of reported bleeding heaviness.

**Table 2:** App-tracked period length (days) for each category of reported period heaviness

Reported period heaviness	N	Length (Mean)	Length (SD)	Length (Median)	Length (Mode)
Very light	199	3.88	1.58	4	3
Light	688	3.98	1.50	4	3
Moderate	3396	4.42	1.38	4	4
Heavy	1595	4.67	1.47	5	4
Very heavy	460	4.96	1.59	5	5

### App-tracked daily flow volume and reported bleeding heaviness

**Fig. 3:**
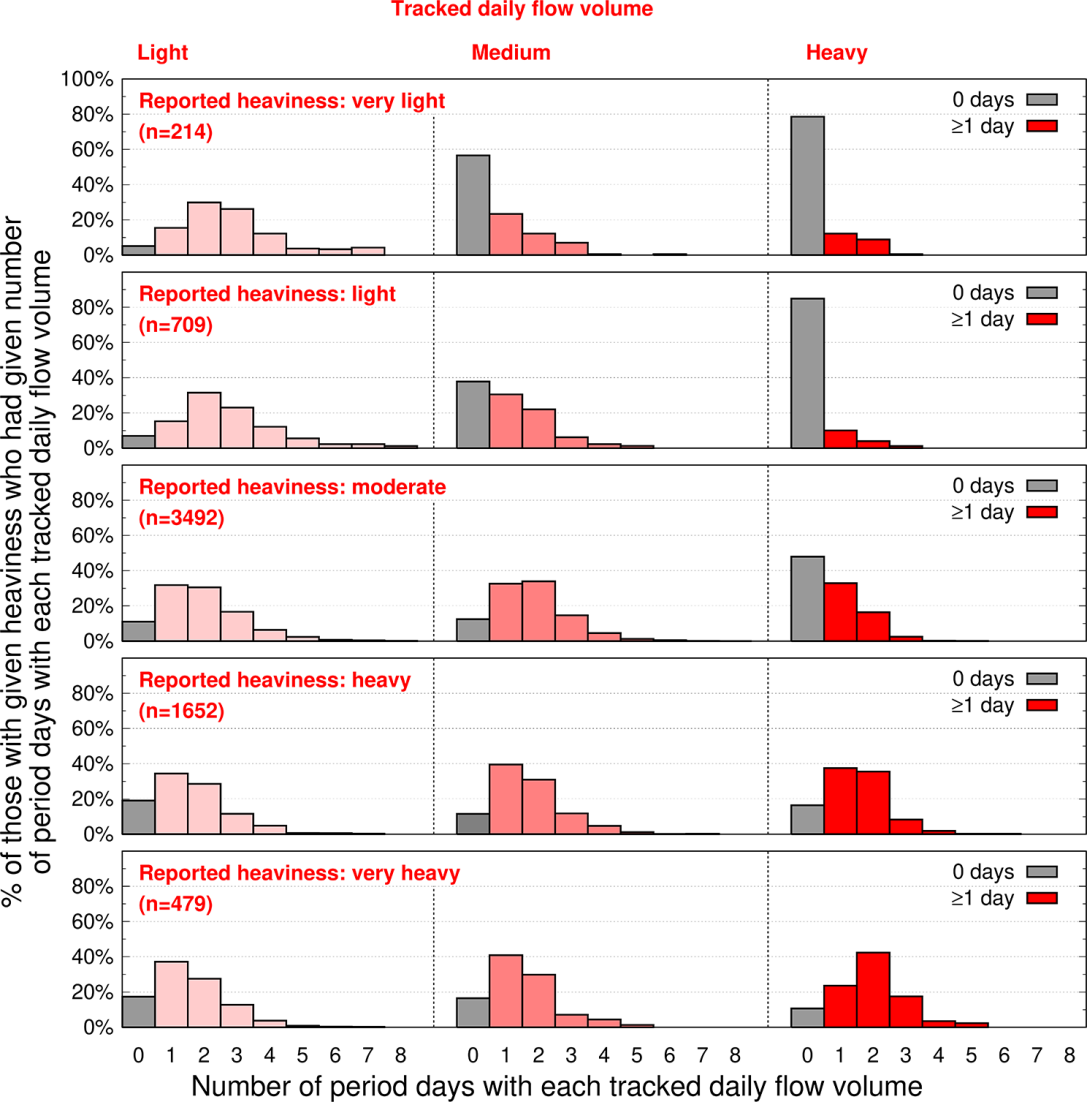
Variability in tracked daily flow volume for each level of reported bleeding heaviness. Each of the five panels is specific to a reported period heaviness: very light, light, moderate; heavy, very heavy (n = number reporting a specific heaviness). Each histogram within a panel is specific to light, medium, or heavy app-tracked days; *x-axis* (for each histogram in each panel): *for each user*, number of app-tracked days that had been tracked as light (histogram 1, pale pink), tracked as medium (histogram 2, pink), or tracked as heavy (histogram 3, red); *y-axis* (for each histogram): % of users in each reported heaviness category; see main text for additional details and an example of interpreting the plotted data

Respondents were asked to characterize the degree of bleeding heaviness during their last period (Question 2; see Methods). For each of the five possible reported characterizations in response to this question (very light, light, moderate, heavy, very heavy), we evaluated variability in the number of days tracked as light, medium, or heavy (Fig. 3). Specifically, for each user, the number of app-tracked days that had been tracked as light, medium, or heavy was tallied (for a given user, the number of days in each of these three tracked categories could be 0 to 8) and contributed to each of the three respective histograms in one of the five panels in Fig. 3. Each histogram within a panel is specific to light, medium, or heavy tracked days; for each histogram, the y-axis is the percent of users with a specific number of tracked days. For example, n = 214 reported their most recent period as “very light” (top panel). Of these, the right-most histogram gives the count of tracked heavy days: ~11% of 214 had tracked 1 day as “heavy”, ~ 8% had tracked 2 days as “heavy”, ~ 1% had tracked 3 days as “heavy”, ~ 80% (grey bar) had not tracked any day as “heavy”.

Several somewhat unexpected patterns emerged from this examination. About 20% of those reporting a very-light/light period had tracked at least 1 heavy day (mode tracked-heavy-day = 1). About 50% of those reporting a moderate period had tracked at least 1 heavy day. More than 50% of those reporting a heavy period had tracked 2 or more heavy days, however, almost 20% had not tracked any heavy days. Similarly, more than 60% of those reporting a very heavy period had tracked at least 2 heavy days, however, ~ 15% had not tracked any heavy days. This seemingly anomalous group (i.e., their reported heaviness appears to be inconsistent with their tracked data) is further examined below. With increasing reported heaviness, mean period length increases from 3.9 to 5.0 days.

### Reported period heaviness and period-associated impairments of wellness

**Fig. 4:**
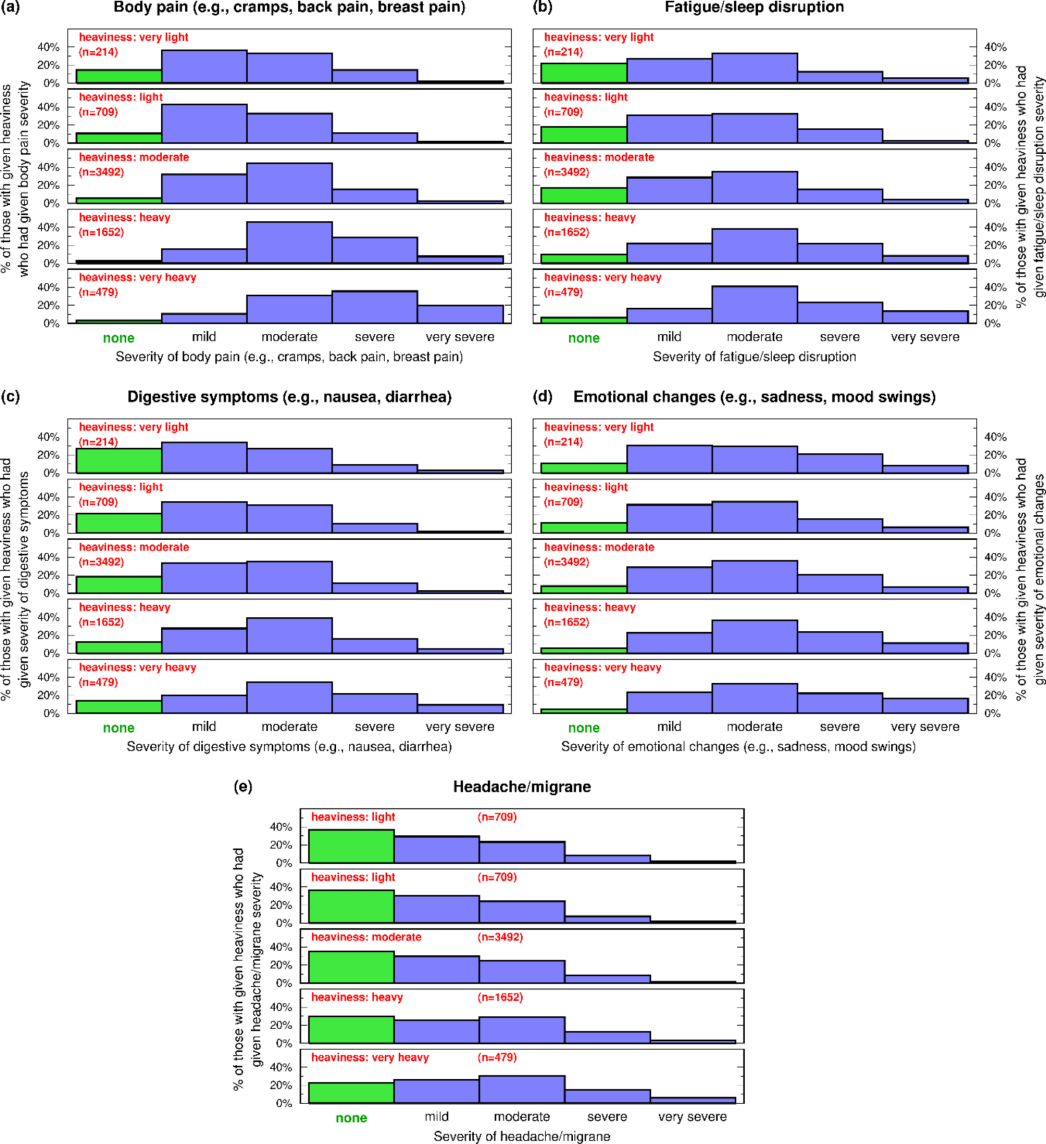
Impact of period heaviness on wellness. The reported severity (*none* through *very severe*) of five impairments [(**a**) body pain, (**b**) sleep problems, (**c**) digestive problems, (**d**) emotional changes, (**e**) headache/migraine] experienced by respondents during their last completed period. For each heaviness category (*very light* to *very heavy*), the distribution of severity for each impairment is plotted; of those with a specified heaviness, green bar = % with none, and blue bars = % with specified severity

Although it is widely recognized that for many women menstrual bleeding is associated with various physical and psychosocial impairments, how these experiences may relate to heaviness is not well understood. For each of five categories of period-associated symptoms, respondents were asked to report the severity (from none to very severe) of their experience during their last period. Histograms of their responses for each level of reported heaviness were constructed (Fig. 4).

Reported impairment severity increased with increasing reported heaviness for some symptoms (body pain, sleep problems, and digestive discomforts). For body pain (histogram set *a*), as heaviness increases from very light to very heavy, the percent of those with no body pain (green bars) decreases, the percent with severe and very severe pain increases, and the modal percent of responses (i.e., most common response for that flow volume) shifts from mild to moderate to severe.

Severity of sleep problems (histogram set *b*) and digestive symptoms (histogram set *c*) both increased with increasing heaviness but changes were more modest than the changes for body pain.

Greater than 90% of all groups reported emotional changes (histogram set *d*), but the severity of changes did not differ markedly with heaviness. However, very severe emotional change was reported by a larger percent of those with very heavy bleeding than in the other heaviness categories.

The severity of headaches/migraines (histogram set *e*) differed little across the bleeding categories, however, the percent reporting no headaches decreased with increasing heaviness.

### Reported period heaviness and period-associated disruptions of daily activities

**Fig. 5:**
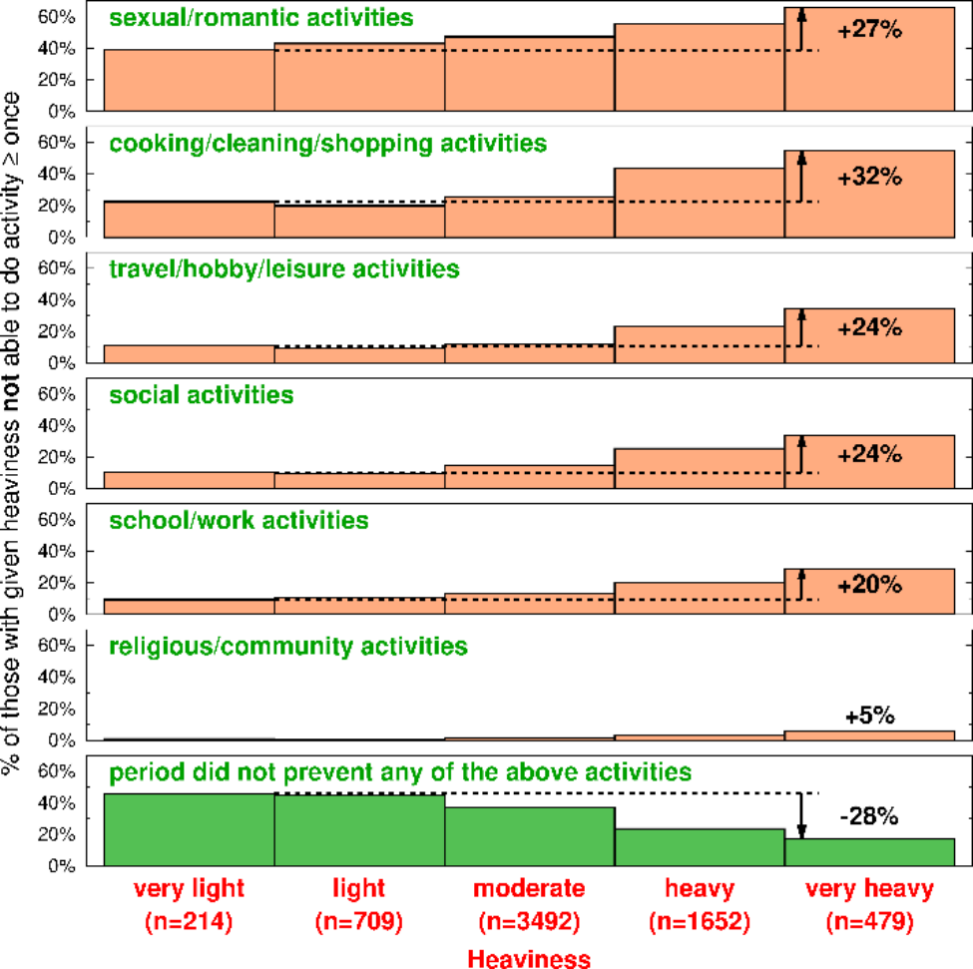
Period-associated disruption of daily activities. Each panel is one of the six activities listed in survey Question 4, plus a bottom panel (in green) representing those persons who were *not* prevented from doing any of the listed activities. The y-axis for each panel is the percent of those persons with a reported heaviness (listed on the x-axis, increasing from left to right) who were not able to do the activity at least once. The increase in percentage who couldn’t do an activity from those with very light flow volume to those with very heavy flow volume is shown in the right-most bar of each panel

As heaviness increased, the percent who couldn’t do a given activity at least once also increased (Fig. 5). Sexual/romantic activities were substantially affected across all degrees of heaviness. Missing religious and community activities differed little across the degrees of heaviness, but this pattern may reflect the generally low level of community engagement during the Covid-19 pandemic. Nearly 50% of those with very light periods were able to do all of the listed activities; less than 20% of those with very heavy bleeding were able to do all of these activities.

### Reported heaviness: more than blood

Those respondents who had reported that their period was either heavy or very heavy were combined into a single sample (“heaviest”) for further investigation. Of these heaviest respondents, those who did not track any heavy days (0HD respondents = 307) were compared to those who had tracked at least one heavy day (≥ 1HD respondents = 1748) to evaluate three post factum hypotheses for the absence of tracked heavy days in the 0HD subsample.

Post factum Hypothesis 1: Perhaps 0HD respondents have longer periods than ≥ 1HD respondents, which could contribute to their experience and reporting of a heavy/very-heavy period even if no single day of their period was heavy.

Post factum Hypothesis 2: Perhaps 0HD respondents did not track all period days. If so, then app-tracked period length is expected to be shorter in 0HD respondents than ≥ 1HD respondents.

Post factum Hypothesis 3: Perhaps period-associated impairments and disruptions are sufficiently severe in 0HD respondents that they experience and report these periods as heavy/very-heavy.

Analyses (Table 3) found that reported mean period length did not differ between 0HD and ≥ 1HD, and app-tracked mean period length was shorter in 0HD than in ≥ 1HD respondents. Both findings were inconsistent with Hypothesis (1) The shorter app-tracked period length in 0HD than in ≥ 1HD respondents was consistent with Hypothesis (2). However, this outcome raises the question of why these respondents would preferentially neglect to track the heaviest day(s). No ready explanation comes to mind. On balance, neither of these two post factum hypotheses were clearly supported.

Analyses of quality of life measures (disrupted activities and severity of period-associated impairments) were consistent with Hypothesis 3. 0HD and ≥ 1HD respondents reported very similar probabilities of experiencing these negative outcomes, and their experiences substantially differed from those reporting lighter heaviness.

These analyses suggest that an individual’s experience and reporting of period “heaviness” is multi-faceted and not only a measure of blood volume per se. This finding is consistent with recent arguments by other scholars [11, 13] and has significant implications for recognizing and treating HMB.

**Table 3:** Statistical tests of post factum hypotheses in the sample of “heaviest” periods

	0 HD respondents	≥ 1 HD respondents	Difference	p
Period Length	(n = 307)	(n = 1748)		t-test
Reported mean (SD)	5.15 (1.35)	5.29 (1.47)	0.14	< 0.1196
App-tracked mean (SD)	4.32 (1.47)	4.81 (1.5)	0.49	< 0.0001
**Disrupted Activities**	**(n = 321)**	**(n = 1810)**		**(Chi-square)**
none of these	25%	22%	3%	0.23
sexual/romantic	56%	58%	2%	0.50
household	45%	46%	1%	0.74
travel/hobby	21%	26%	5%	0.06
social	24%	28%	4%	0.14
school/work	18%	23%	5%	0.05
religious	3%	4%	1%	0.39
**Wellness Impairments**	**% severe + very severe**	**% severe + very severe**		
body pain	43%	40%	3%	0.31
fatigue/sleep disruption	33%	31%	2%	0.48
digestive problems	23%	23%	0%	--
emotional changes	40%	35%	5%	0.09
headaches/migraines	20%	17%	3%	0.19

HD = heavy days; SD = standard deviation; heaviest periods = heavy and very heavy periods

## Discussion

Estimates of the prevalence of HMB vary several fold, in part because commonly used medical criteria are difficult to assess in a representative sample of a population. This obstacle aside, a single or suite of criteria that focus solely or principally on menstrual blood volume for identifying HMB may not adequately capture many individuals’ multi-faceted menstrual bleeding experience.

The findings from this study support the hypothesis that respondents’ reports of heaviness are based on both bleeding volume and other salient attributes of their periods. Experiencing severe period-associated impairments and disruptions contribute to respondents’ characterizations of their periods as heavy or very heavy even when they’ve not tracked any days of heavy bleeding. Period length may also contribute to the respondents’ assessments of period heaviness. As period length increased, the percent of respondents describing their period as heavy or very heavy increased. With increasing reported heaviness, mode and mean period length increased from 3 to 5 days and 3.9 to 5.0 days, respectively. These observations and conclusions are consistent with similar arguments made by others [11, 13].

Period-associated disruption of activities were experienced across all degrees of heaviness, affecting nearly half of those with very light periods and more than 80% of those with very heavy flow volume. Notably, sexual/romantic activities are affected by about 40% of those with even very light periods. This pattern may reflect avoiding intercourse during any level of period bleeding for a range of reasons including cultural or religious prohibitions, personal or partner preferences, and/or period associated impairments that interfere with intercourse [30]. Clearly, quality of life can be affected by all bleeding levels and should be addressed regardless of reported flow volumes.

When asked about their most recent period, 83% of respondents reported a period length to within 1 day of the number of days tracked in the app. However, only 44% of respondents were able to recall the exact number of bleeding days. The difference between questionnaire data compared to app-tracked data is most likely attributable to recall bias. Additionally, we found that experienced period duration appears to influence respondent’s reported bleeding duration. Those with shorter period lengths (as tracked in the app) were more likely to overestimate their number of bleeding days in the questionnaire while those with longer periods were more likely to underestimate their period length, a pattern which could contribute to under-diagnosis of HMB.

Cultural beliefs regarding normal cycle length and period duration may also influence an individual’s self-reporting. Typical representations of a menstrual cycle generally describe a 28-day long cycle with ovulation happening mid-cycle and an average of 5 bleeding days [20]. The influence of these cultural norms may unconsciously influence an individual’s recollection their own cycle attributes.

Our findings corroborate those from other studies that have investigated the differences between recalled cycle lengths and cycle lengths tracked in daily diaries. These studies showed a tendency to report 28-day and 30-day cycles, even though the actual cycle lengths tracked in daily diaries differed from these canonical cycle lengths [31, 32]. Users with relatively shorter or longer mean cycle lengths were more likely to be inaccurate in their self-reporting and inaccuracy increased with cycle variability [32]. The potential impact of cultural perceptions of the menstrual cycle on individuals’ recall of their bleeding patterns illustrates the utility of app-tracked data to minimize this type of bias when collecting bleeding history.

In this study, reported heaviness was associated with app-tracked period length: the shorter the period, the more likely respondents were to report their last period as light or very light, while longer app-tracked periods (6 to 10 days) were most commonly reported as very heavy or heavy. Similarly, reported heaviness was associated with the number of app-tracked heavy days: respondents who reported their last period as very heavy or heavy had tracked on average at least 1.5 days of heavy bleeding, while respondents reporting a very light or light period had on average tracked less than half a day of heavy bleeding. However, some respondents with short periods still reported heaviness while a fraction of respondents who had tracked over 6 days of bleeding reported their last period as light. This suggests that period length and the number of heavy days are only two components of the characterization of period heaviness.

The increasing popularity of menstrual tracking apps presents an opportunity to incorporate a new data source into the menstrual health clinical assessment toolkit, notably in the evaluation of HMB. Our results highlight the utility of app-tracked data for documenting bleeding histories and support a growing consensus among menstrual health researchers regarding the need for data collection tools that are more sensitive to a wide range of individual bleeding experiences.

### Study advantages

Study advantages include a very large non-clinical sample that spanned the adult reproductive years (18–45) and was representative of the ethnic diversity in the U.S., and a questionnaire that could easily be taken in Spanish as well as English. In addition, flow volume categories in the questionnaire were similar to those used in the app, therefore, differences between app-tracked flow volume and reported flow volume are likely to be an outcome of the multi-faceted assessment of the period that was experienced by the user rather than an artifact of disparate terminology. Of particular importance, all study participants had been using the Clue app prior to completing the questionnaire, thus eliminating differences that could arise if participants had been using different menstrual tracking apps. Also, use of a tracking app may have helped study participants to be more consciously aware of their own menstrual experiences, and thus better able to accurately report those experiences, than would be the case for persons not using any tracking app.

### Study limitations

Limitations of this study include the younger average age of adult Clue users compared to the general U.S. population. However, this difference may not be important for our reported findings because in the present study there were no significant differences in the responses of younger (ages 18–25 years) and older (ages 26–45 years) participants.

Because study participants were 18 years and older, the findings may not be generalizable to younger age groups, therefore further study is needed on the HMB experiences of younger persons.

We were also limited in the number of activity options listed in Question 4. For example, we did not list “sports/exercise” as a category separate from “leisure activities”.

This study assumed that app-tracked data were an accurate real-time record of experienced menstrual bleeding. “App fatigue” refers to low or inconsistent engagement with apps or other related tools, with usage fatigue being one reason (among many) for the low engagement [33]. This behavior could result in poor data quality if someone is not tracking symptoms because they are no longer engaging with the app rather than because they are not experiencing the symptoms they had previously tracked. Although app users can and do forget to log data or may log on some subsequent day than the day on which bleeding occurred, the analyses in our study suggest data omission was not common. This potential problem was mitigated at the outset by not including users who had tracked only one day of bleeding (fatigue could have been a reason not to track the subsequent bleeding days). Furthermore, we selected for persons who tracked within a certain timeframe, were active within the app, and filled out a (relatively long) survey questionnaire. These persons are therefore likely to be more engaged users. This inference is supported by the statistical similarity of tracked and reported variables. Tracked and reported period length were identical for 44% of the respondents and differed by only 1 day for an additional 39% of the respondents. In addition, this study sample’s descriptive statistics for cycle length (calculated from the app-tracked period data entered by the user) were consistent with published data on cycle length in U.S. women. This finding bolsters the assumption that most users of menstrual health tracking apps (or at least, most Clue users) accurately tracked their days of menstrual bleeding. On the other hand, because of their apparently high engagement, our study population may not be reflective of all app users (i.e., more engaged users may have different characteristics than less engaged users).

The flow volume levels (light, medium, heavy) in this app (and, to the best of our knowledge, in any app) have not been validated against an independent measure of flow volume. These descriptors are interpreted by the user as best fits their own needs and experiences, and the exact meaning undoubtedly differs across users. On the other hand, the observed consistency of several patterns in the data (e.g., severity of impairments increases with increasing flow volume) supports the reasonable expectation that there is also some shared meaning across users, at least among U.S. Clue app-trackers, of these flow volume descriptors.

In addition, respondents were not instructed on whether or not to check their app-tracked data as they completed the questionnaire, therefore their recalled data may be more accurate than would be the case without immediate access to such records. However, Clue user testing of other questionnaires has observed that respondents do not commonly attempt to switch screens to their tracked data because of a concern that they will not be able to switch back to the questionnaire screen. In any case, respondents’ greater awareness and knowledge of their cycles does not necessarily reduce the generalizability of our findings regarding the multi-faceted experience of menstrual bleeding.

## Conclusions

This study is the first to compare real-time bleeding data tracked in a menstrual health app by a non-clinical sample of more than 6500 persons to subsequently reported (questionnaire) data matched to the same persons. A large majority of reported period length durations were within 1 day of the tracked data, however, those with the longest periods were more likely to underestimate their period duration, a pattern that could contribute to under-recognition of HMB.

Our analyses revealed considerable variability in individuals’ characterizations of their bleeding experience. Period heaviness is a complex construct that encapsulates flow volume and, for many, several other bleeding-associated experiences (period length, bodily impairments, disruptions of daily activities). Even very precise flow volume assessments cannot capture the multi-faceted nature of HMB as experienced by the individual. These findings strongly support the inclusion of individual experiences in the clinical assessment of HMB. Real-time app-tracking facilitates quick daily recording of several aspects of bleeding-associated experiences. We anticipate that menstrual tracking can support more comprehensive and individualized assessments of HMB in clinical settings by affording users greater ownership over their bleeding data, a better understanding of their individual patterns, and the ability to share more detailed information with their clinicians.

## Electronic supplementary material

Below is the link to the electronic supplementary material.

**Additional File:** Table A1: sample’s age distribution; sample’s education distribution; Table A2: sample’s race/ethnicity distribution compared to 2020 US Census.

## Data Availability

The datasets used and/or analysed during the current study are available at the Dryad research data repository <https://doi.org/10.5061/dryad.tmpg4f52w>.

## Abbreviations

HMB: Heavy menstrual bleeding

## Notes

The original online version of this article was revised: the figure 3 has been updated.
