# Correction: More than blood: app-tracking reveals variability in heavy menstrual bleeding construct

**DOI:** 10.1186/s12905-023-02352-w

**Published:** 2023-04-25

**Authors:** Amanda A. Shea, Fiorella Wever, Cécile Ventola, Jonathan Thornburg, Virginia J. Vitzthum

**Affiliations:** 1 Clue By BioWink GmbH, Adalberstrasse 7-8, 10999 Berlin, Germany; 2 University of Amsterdam, Amsterdam, Netherlands; 3 Indiana University, Bloomington, IN USA

**Correction: BMC Womens Health 23, 170 (2023)**

**https://doi.org/10.1186/s12905-023-02312-4**

Following publication of the original article [1], in this article the Fig. 3, should have appeared as shown below:

**Fig. 3:**
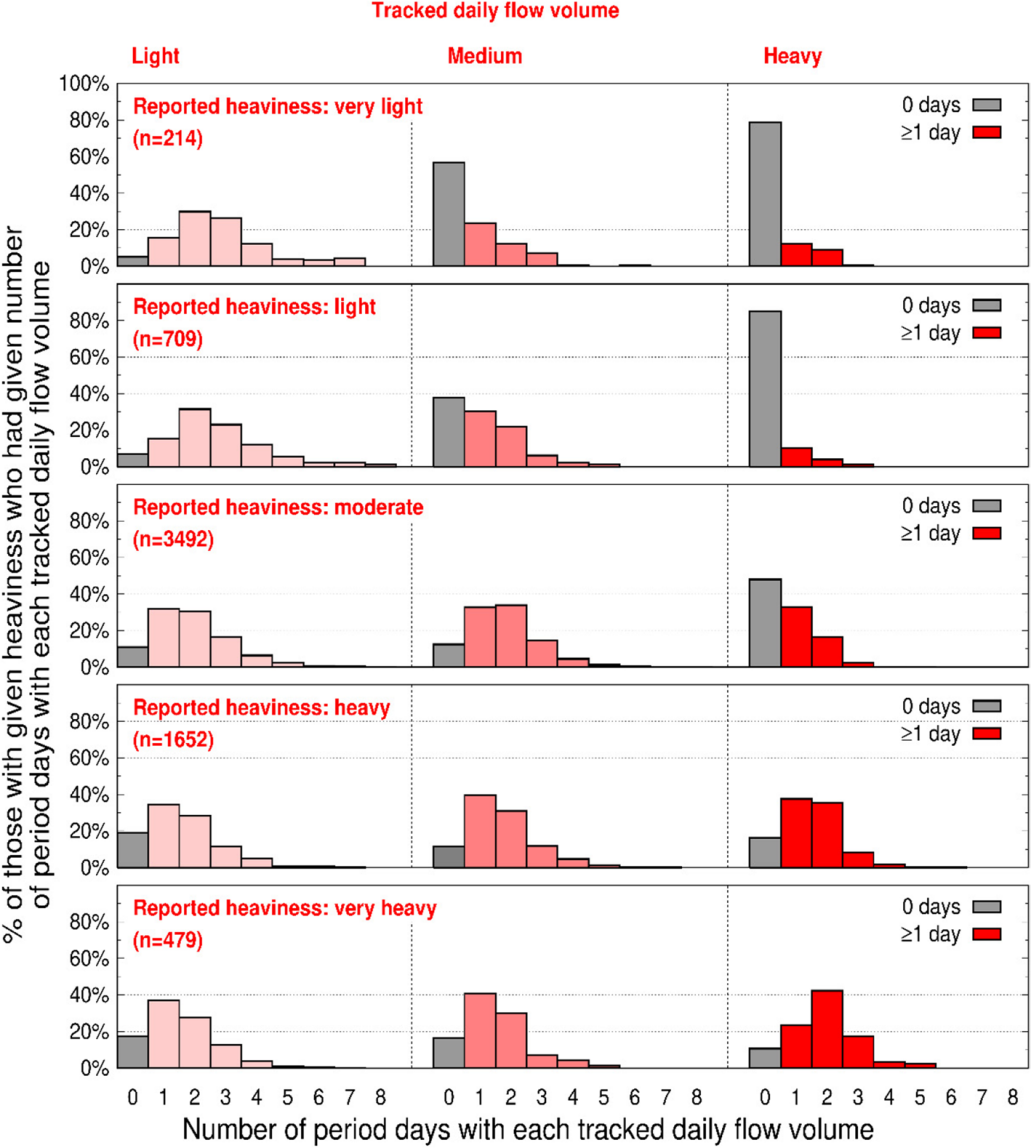
Variability in tracked daily flow volume for each level of reported bleeding heaviness. Each of the five panels is specific to a reported period heaviness: very light, light, moderate; heavy, very heavy (n = number reporting a specific heaviness). Each histogram within a panel is specific to light, medium, or heavy app-tracked days; *x-axis* (for each histogram in each panel): *for each user*, number of app-tracked days that had been tracked as light (histogram 1, pale pink), tracked as medium (histogram 2, pink), or tracked as heavy (histogram 3, red); *y-axis* (for each histogram): % of users in each reported heaviness category; see main text for additional details and an example of interpreting the plotted data

The original article has been corrected.
