# Supplementary material for: More than blood: app-tracking reveals variability in heavy menstrual bleeding construct
Source: BMC Womens Health. 2023 Apr 11;23:170. doi: 10.1186/s12905-023-02312-4 (PMC10088691; doi:10.1186/s12905-023-02312-4)
Supplement: Supplementary file 1 — Additional File: Table A1: sample’s age distribution; sample’s education distribution; Table A2: sample’s race/ethnicity distribution compared to 2020 US Census. [file 12905_2023_2312_MOESM1_ESM.pdf]

Additional Material Provided

Filename: Additional_file_1.pdf

File format: .pdf

Title: Demographic statistics of study sample

Description:

Table A1: sample's age distribution; sample's education distribution;

Table A2: sample's race/ethnicity distribution compared to 2020 US Census.

Table A1: Study Participant Demographics (total n=8051)

Age (years)	N (% of 6546)
18-19	656 (10%)
20-24	2135 (33%)
25-29	1610 (25%)
30-34	1187 (18%)
35+	958 (14%)
Education level	N (% of 6726)
No formal education	3 (< 0.5%)
Primary education (grade school)	17(< 0.5%)
Secondary education or equivalent (high school)	1970 (27%)
Tertiary education (at least some post-secondary)	4726 (72%)
Not answered	10 (< 0.5%)

Table A2: Race/Ethnicity of Study Sample compared to 2020 U.S. Census

Source for census data: <https://www.census.gov/quickfacts/fact/table/US/PST045221>

Respondent Selected	This Study (N)	This Study (% of 6546)	US 2020 census
White only	4150	63.4	60.1
White Hispanic	259	4.0	NA
	4409	67.4	76.3
Black only	464	7.1	NA
Black Hispanic	69	0.9	NA
	533	7.9	13.4
Asian only	290	4.4	NA
Asian Hispanic	9	0.1	NA
	299	4.6	5.9
Native only	15	0.2	NA
Native Hispanic	15	0.2	NA
	30	0.5	1.3
Hawaii only	4	0.06	NA
Hawaii Hispanic	3	0.05	NA
	7	0.1	0.2
Total of above	5278	80.4	97.1
Multi-race (two or more races)	379	5.8	NA
Multi-race Hispanic	72	1.1	NA
	451	6.9	2.8
Hispanic only	740	11.3	NA
No Answer or "other"	77	1.2	NA
TOTAL	6546	99.8	99.9
All Hispanic	1167	17.8	18.5
